# Molecular and genetic analysis of defensive responses of *Brassica juncea* – *B. fruticulosa* introgression lines to *Sclerotinia* infection

**DOI:** 10.1038/s41598-019-53444-3

**Published:** 2019-11-19

**Authors:** Chhaya Atri, Javed Akhatar, Mehak Gupta, Neha Gupta, Anna Goyal, Kusum Rana, Rimaljeet Kaur, Meenakshi Mittal, Anju Sharma, Mohini Prabha Singh, Prabhjodh S. Sandhu, Martin J. Barbetti, Surinder S. Banga

**Affiliations:** 1 DBT Centre of Excellence on Brassicas, Department of Plant Breeding and Genetics, Punjab Agricultural University, Ludhiana, 141004 Punjab India; 2 School of Agriculture and Environment and the UWA Institute of Agriculture, Faculty of Science, The University of Western Australia, 35 Stirling Highway, Crawley, WA 6009 Australia

**Keywords:** Agricultural genetics, Environmental impact

## Abstract

*Sclerotinia* stem rot caused by *Sclerotinia sclerotiorum* is a major disease of crop brassicas, with inadequate variation for resistance in primary gene pools. We utilized a wild *Brassicaceae* species with excellent resistance against stem rot to develop a set of *B. juncea - B. fruticulosa* introgression lines (ILs). These were assessed for resistance using a highly reproducible stem inoculation technique against a virulent pathogen isolate. Over 40% of ILs showed higher levels of resistance. IL-43, IL-175, IL-215, IL-223 and IL-277 were most resistant ILs over three crop seasons. Sequence reads (21x) from the three most diverse ILs were then used to create *B. juncea* pseudomolecules, by replacing SNPs of reference *B. juncea* with those of re-sequenced ILs. Genotyping by sequencing (GBS) was also carried out for 88 ILs. Resultant sequence tags were then mapped on to the *B. juncea* pseudomolecules, and SNP genotypes prepared for each IL. Genome wide association studies helped to map resistance responses to stem rot. A total of 13 significant loci were identified on seven *B. juncea* chromosomes (A01, A03, A04, A05, A08, A09 and B05). Annotation of the genomic region around identified SNPs allowed identification of 20 candidate genes belonging to major disease resistance protein families, including *TIR-NBS-LRR* class, Chitinase, Malectin/receptor-like protein kinase, defensin-like (*DEFL*), desulfoglucosinolate sulfotransferase protein and lipoxygenase. A majority of the significant SNPs could be validated using whole genome sequences (21x) from five advanced generation lines being bred for *Sclerotinia* resistance as compared to three susceptible *B. juncea* germplasm lines. Our findings not only provide critical new understanding of the defensive pathway of *B. fruticulosa* resistance, but will also enable development of marker candidates for assisted transfer of introgressed resistant loci in to agronomically superior cultivars of crop *Brassica*.

## Introduction

*Sclerotinia sclerotiorum*, causing *Sclerotinia* stem rot, is a serious pathogen of oilseed crops including *Brassica juncea* (Indian mustard)^1–3^. This necrotrophic fungus attacks mustard crops at all crop growth stages, especially at flowering, causing yield losses of 10–90% and impaired oil quality^3,4^. Mustard is an important oil seed crop of India and worldwide^4,5^, but faces increasing challenges to its productivity from this pathogen. Moreover, wide host range of *S. sclerotiorum* and the long period of survival of its resting sclerotia together limit the option for control through crop rotation or tillage. Fungicide application, at best, provides inconsistent control and is expensive. Hence there is a need to better understand host-pathogen interactions for using host resistance. A plethora of pathogenicity factors; mainly oxalic acid^6,7^, hydrolases and poly-galacturonases^8^, small RNAs^9^ have been reported to facilitate *Sclerotinia* infection and colonization on rapeseed-mustard, soybean, pea and sunflower. These pathogen-associated molecular patterns act as the initial trigger for altered redox homeostasis of host tissue and switches on the necrotrophic mode of stem rot pathogen after oxidative burst^10^.

The only sustainable and economical way to manage *Sclerotinia* stem rot is via genetic improvement to enhance inherent resistance in the host through utilization of conventional/molecular breeding approaches.

Unfortunately, the complex genetic basis of resistance poses challenges in conventional breeding due to involvement of diverse signalling factors (transcription factors, hormones, enzymes, proteins etc.) associated with the host-pathogen interaction^11–13^. Partial resistance to *S. sclerotiorum* has been reported in some Chinese^1,14^ and Australian^1,15^ lines of oilseed rape, but complete genetic resistance to this pathogen does not occur in crop brassicas. However, high levels of disease resistances are present in genetically diverse wild allies of the family *Brassicaceae* such as *Erucastrum gallicum*, *Capsella bursa*-*pastoris*, *E*. *cardaminoides*, *Diplotaxis tenuisiliqua*, *B*. *fruticulosa* and *B*. *oleracea*. Many of these have been utilized in recent research into stem rot resistance in *B. napus* and *B. juncea*^16–18^. In addition, a number of QTL mapping studies with biparental mapping population have helped to identify chromosomal loci linked to *Sclerotinia* resistance in *B. napus*^19–22^, soybean^23^ and sunflower^24^. Integration analysis of previously known QTLs of the *B. napus* genome have allowed identification of conserved QTLs and clusters of nucleotide-binding-site, leucine-rich-repeat-containing candidate genes for *Sclerotinia* resistance on chromosomes A09 and C06^25^. However, low mapping resolution due to limited recombination events and allelic diversity of these QTL mapping studies constrained prediction of candidate gene(s) and development of marker assisted selection for disease resistance. Fortunately, genome-wide association studies (GWAS) allows improved mapping resolution by exploiting historical recombination events and high allelic diversity present in association panels^26,27^. Further, GWAS approaches are more cost effective for the identification of the minor QTLs with small effects that underlie many complex traits such as disease resistance^20,28–31^. A few success stories for candidate gene association with host infection as well as resistance have been reported in soybean^13,32–36^ and *B. napus*^37,38^ through SNP markers validation by c-DNA microarrays^39–42^, RT-seq^43^ and proteomic^44^ approaches. GWAS have also revealed association of significant SNPs on chromosomes A08, C04, C06 and C08 in *B. napus* to *Sclerotinia* stem rot resistance. These SNPs are close to genes involved in oxidative burst, lignin biosynthesis and jasmonic acid pathway^37,38^.

In our previous study^18^, we demonstrated significant stem rot resistance through introgression of *B. fruticulosa* genomic fragments in to *B. juncea*. Molecular characterisation of developed ILs was carried out through SSR based linkage mapping and cytogenetic tools^18^. Although, ten significant marker-trait associations to disease resistance of ILs were discovered consistently over two years of study, there were large mapping intervals as a limited number of transferable SSR markers did not permit precise localization of resistance associated genes. To facilitate finer resolution in search of candidate genes, we now report extension of our previous research studies^18^through GBSof 88 ILs. This generated large numbers of marker trait associations (MTA’s) and highlighted 49 significant SNPs corresponding to thirteen loci on chromosomes A01, A03, A04, A05, A08, A09 and B05. Many of these MTA’s were consistent across crop seasons and explained significant phenotypic variation (7.34% to 16.04%), and helped to predict 20 candidate genes belonging to major disease resistance protein families. We discuss how discovery of such novel SNP marker associations to *Sclerotinia* stem rot resistance will not only facilitate novel candidates contributing to mustard immune response, but will also enable development of breeder-friendly markers for assisted introgression of resistant loci into agronomically superior cultivars of crop *Brassica *against *Sclerotinia* stem rot.

## Results

### Phenotypic evaluation of the *Sclerotinia* stem rot resistance

**Figure 1:**
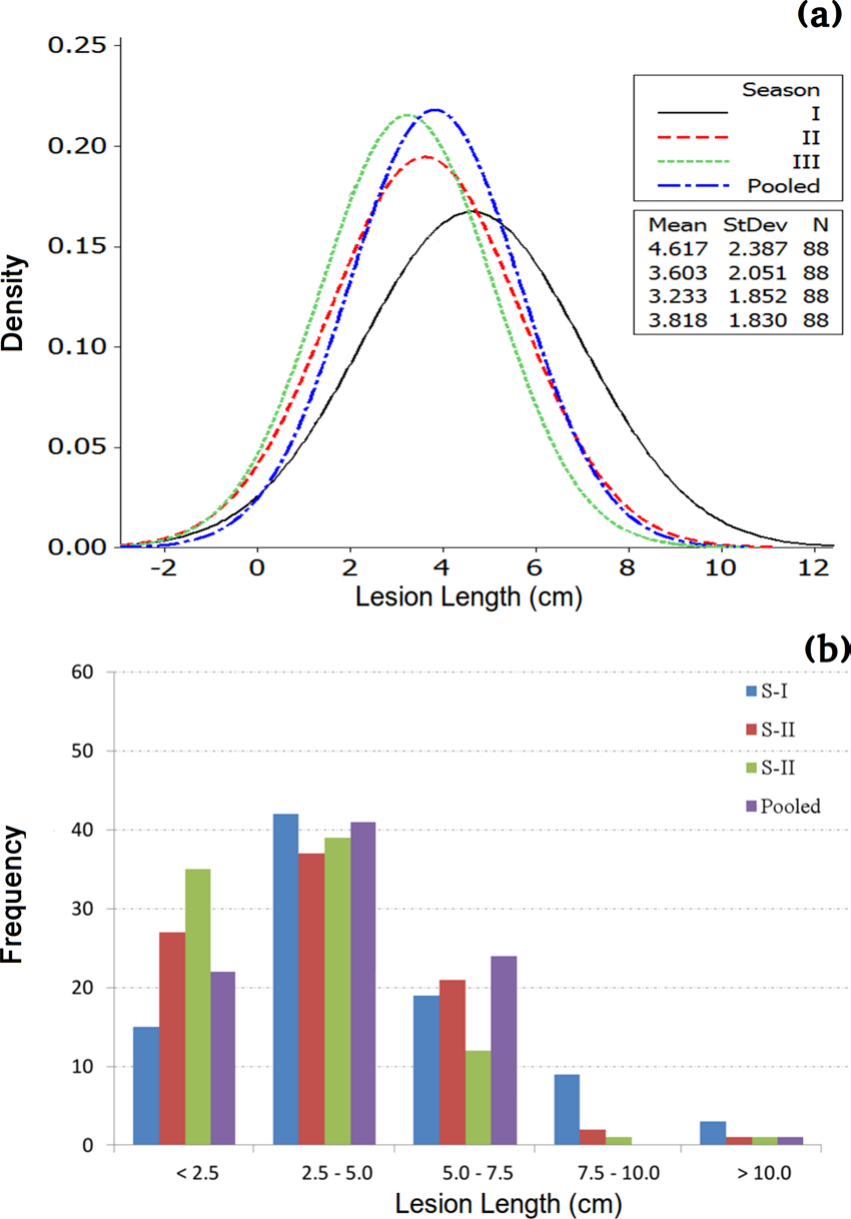
(**a**,**b**): Phenotypic distributions and frequency histogram of stem lesion length in *Brassica juncea* – *B. fruticulosa* intro in season I (2012–13), season II (2013–14), season III (2016–17) and pooled across the three seasons.

**Figure 2:**
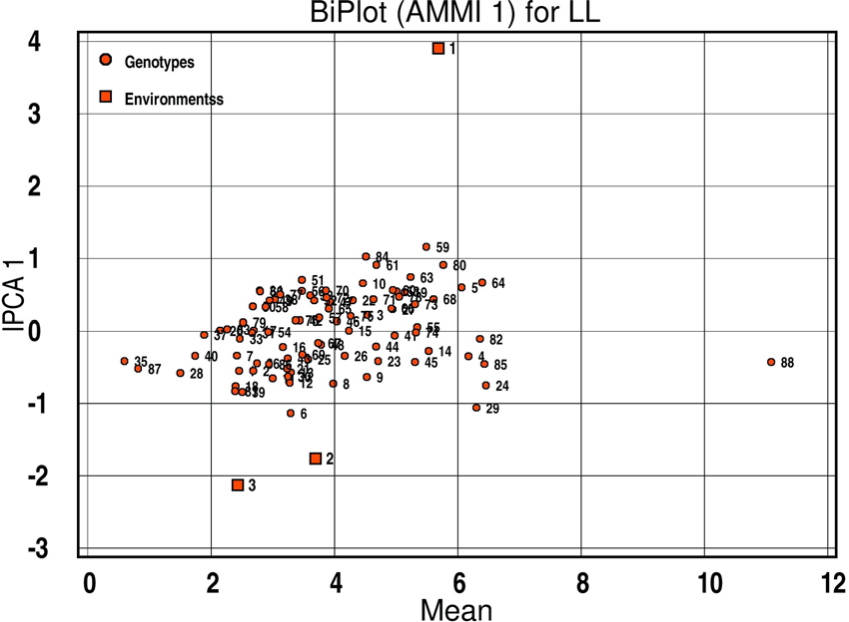
AMMI biplot analysis of 88 *Brassica juncea* – *B. fruticulosa* introgression lines across three environments.

Phenotypic data for 2011–12 and 2014–15 was the same as published earlier^18^.  Screening of ILs was extended for another year during 2017–18. Continuous phenotypic variations for stem rot resistance confirmed the quantitative nature of *Sclerotinia* stem rot resistance in *B. juncea* - *B. fruticulosa* ILs. Frequency and disease score distribution graphs of 86 introgression lines along with susceptible and resistant checks are presented in Fig. 1(a,b). Analysis of variance (ANOVA) revealed significant differences among the ILs in terms of expression of resistance responses to stem inoculation (Table 1). Variation across years and year × genotype (G × E) effects were also significant. Replication effects were non-significant. Mean values for stem lesion length were: 4.62 cm (season I), 3.60 cm (season II) and 13.23 cm (season III). The corresponding values for the susceptible genotype RLC1 and resistance donor *B. fruticulosa* were 11.2, 11.8, 10.38, 11.13 and 0.60, 0.30, 1.53, 0.81 cm of lesion length during seasons I, II and III and pooled, respectively. Lesion length scores differed over seasons. Of the ILs investigated, 57 lines fell under the resistant category in season I, 65 in season II and 74 in season III, respectively. Among these, 40% of genotypes were highly resistant with mean lesion length < 2.5 cm during season III, whereas in seasons I and II, proportions of such genotypes were 17% and 31%, respectively (Fig. 1b). AMMI analysis also confirmed variation across environment and genotype × year interactions (Fig. 2). Season II had low interaction, while season I and season III were highly interactive. Season I was rated as a favourable environment but season III was considered an unfavourable environment for disease built up. Out of 88 genotypes, 83 genotypes had lower year × genotype effects and hence they were stable in their resistance responses. Five ILs, namely IL-43, IL-175, IL-215, IL-223 and IL-277, were consistent for their highly resistant responses to stem inoculation over three years of testing.

**Table 1:** ANOVA result for trait stem lesion length in *Brassica juncea - B. fruticulosa* introgression lines.

Source	df	Sum of Squares	Mean Square
Year	2	950.33	475.16***
Replication	1	1.13	1.13
Genotype	87	1212.59	13.94***
Year × Genotype	174	1618.01	9.30**
Year × Replication	2	15.83	7.92
Replication × Genotype	87	410.09	4.71
Error	174	996.07	5.72
Total	528	13416.14	

Significance: ** < 0.01 and *** < 0.001 p-value.

### Identification of MTA’s using GWAS

**Figure 3:**
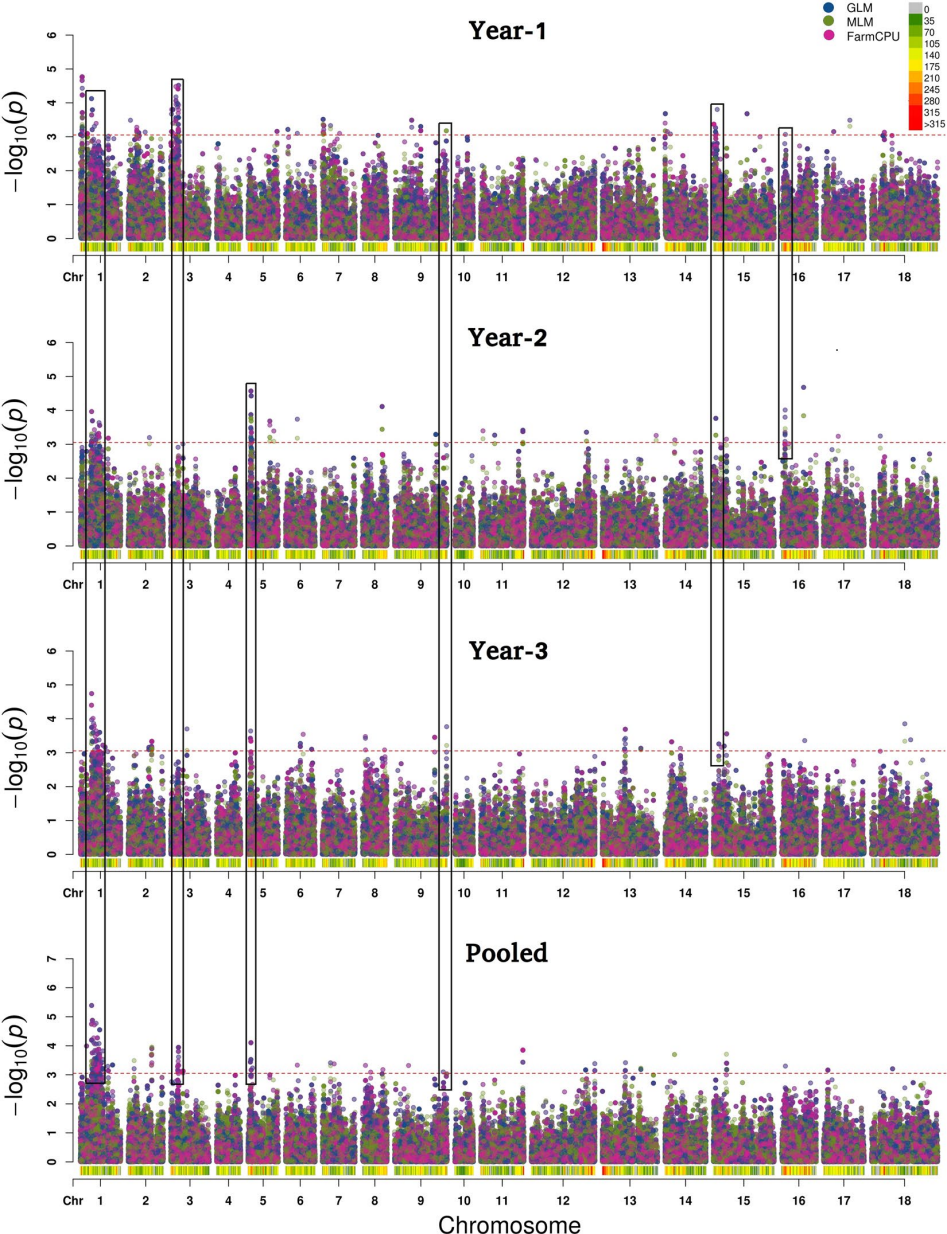
Manhattan plots showing GWAS outcomes for *Sclerotinia sclerotiorum* stem lesion length.

**Figure 4:**
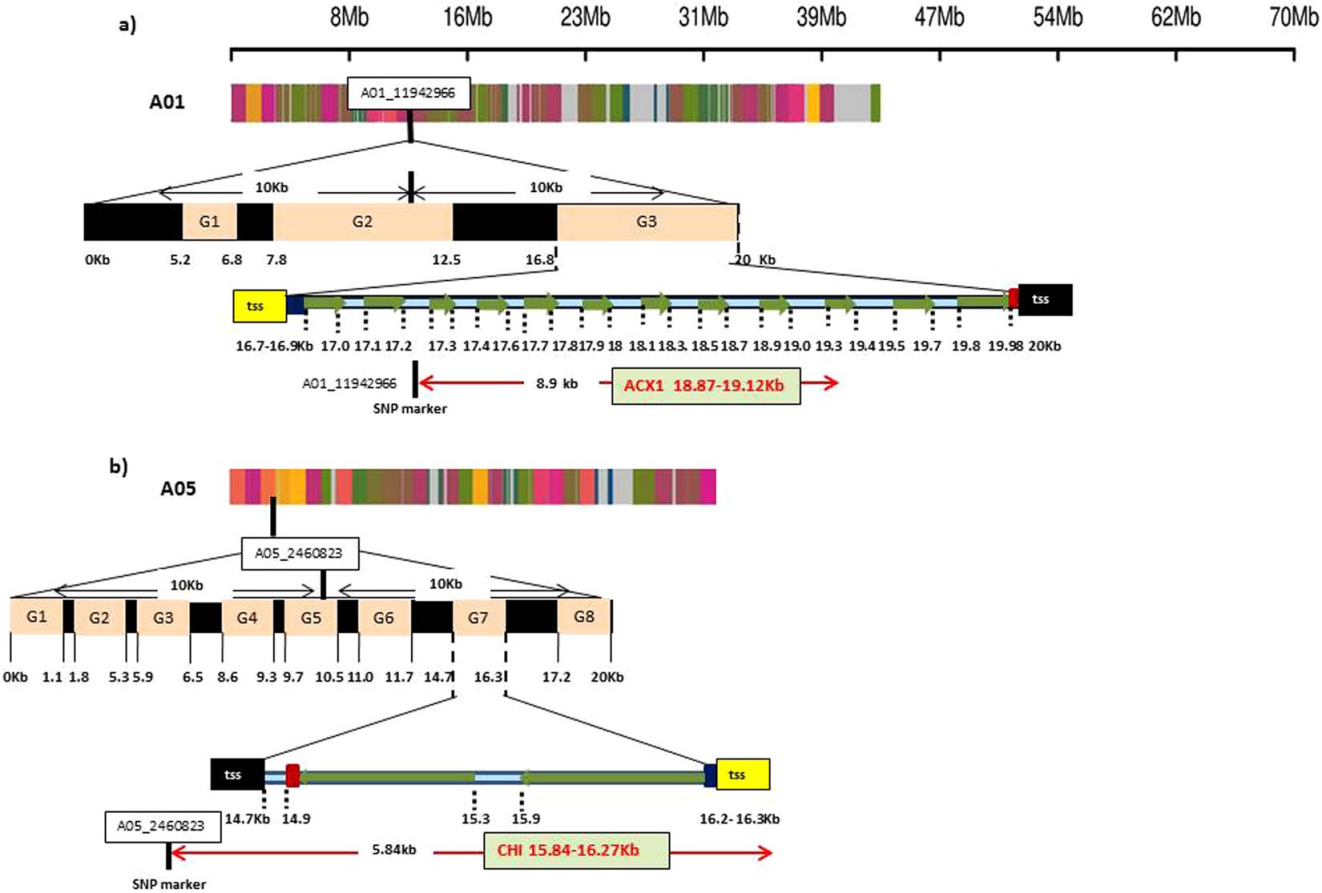
Position of candidate genes near SNP marker spanning 20 kb genomic region on chromosomes (**a**) A01 (**b**) A05 of *B. juncea*. Genes are predicted in 20Kb region using AUGUSTUS tool.

**Figure 5:**
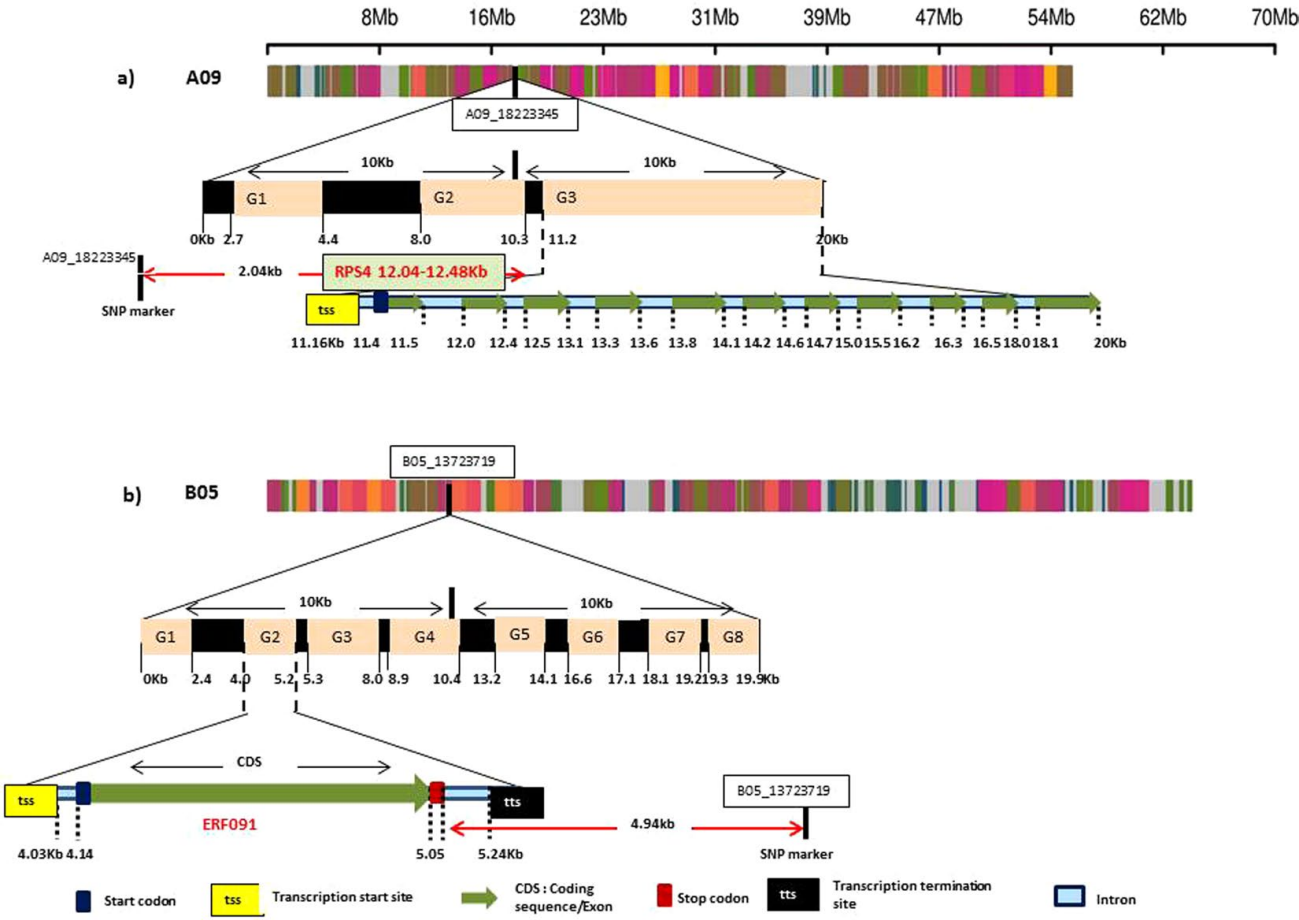
Position of candidate genes near SNP marker spanning 20 kb genomic region on chromosomes (**a**) A09b) B05 of *B. juncea*. Genes are predicted in 20Kb region using AUGUSTUS tool.

Genotyping by sequencing allowed identification of 31,896,811 SNPs. Only 88,624 SNPs were retained after discarding low quality SNPs and eliminating the ones with minor allele frequency <0.08. These were spread across 18 chromosomes (Supplementary Fig. S1). Heat map of kinship matrix showing genetic relatedness among 88ILs is presented in Supplementary Fig. S2. Horizontal lines on top of heat map show hierarchical clustering of ILs. There were two broad groups, the first group was less diverse and ILs included in this group showed consistently superior resistance responses. Discriminant Analysis of Principal Components (DAPC), however, revealed three major groups (Supplementary Fig. S3). GWAS was performed using five models [GLM, MLM, FarmCPU, GLM(T) and MLM(T)] simultaneously. Goodness of fit of GWAS models was implemented using Quantile-Quantile (Q-Q) plots (Supplementary Fig. S4). We retained only those SNPs that were repeatedly detected over at least two algorithms. Also included were the MTA’s that were consistently detected over at least two seasons. All the SNPs showing strong LD with each other were clumped using LD block as a criterion to define a major QTL. After adopting the threshold -log10(*p*) value (>3) and clumping of SNPs, thirteen significant loci scattered across seven chromosomes (A01, A03, A04, A05, A08, A09 and B05) were considered (Table 2). Manhattan plots were generated with multi-model plotting (Fig. 3). A01 had the greatest number of five significant loci, followed by three loci on A05 and only one each on A03, A04, A08, A09 and B05. The phenotypic variation explained by these loci ranged from 7.34% to 16.04%. Amongst five peak associated loci detected on chromosome A01 genomic region, A01_11942966-4403 included a cluster of seven SNPs that were in full linkage disequilibrium (LD). These covered a genomic interval of 1437 bp. This lead association signal explained 11.75% of phenotypic variation. Annotation was suggestive of the gene *ACX1* in close vicinity (Fig. 4a). This gene encodes acyl CoA oxidase 1 protein. ASNP A01_12606026 was mapped close to the gene *LOX-5*, encoding Lipoxygenase-1 protein. SNP A01_15152306 was also associated with a similar gene (*LOX-1*), belonging to the *PLAT/LH2* domain-containing lipoxygenase family. A group of five SNPs (A01_23456622-875) clustered in a small genomic region (253 bp) was also identified on A01 with indicative association with *SOT18* that encodes desulfoglucosinolate sulfotransferase protein. A SNP on A01 (A01_23756215) explained 8.18% of phenotypic variation. The nearest gene to this MTA was *At1g34047*, which encodes defensin-like family protein. Single SNP locus identified on chromosome A03 was recorded close to *PHOS32* encoding adenine nucleotide alpha hydrolases-like superfamily protein. Also present, at a short genomic distance, was the gene *THE1*. This gene encodes a Malectin/receptor-like protein kinase family protein. A group of 13 SNPs on chromosome A04 (A04_19791849-2116), covering a small genomic region of 267 bp were present close to the gene *MPKKK17*. Out of four associated loci, detected on chromosome A05, one locus comprising five SNPs (A05_2460823-1061) and spanning a genomic region of only 238 bp was located adjacent to *AT2G43620* and *CHI* encoding proteins belonging to the chitinase family (Fig. 4b). A genome space (A05_3029963-50244), carrying seven SNPs was also located on chromosome A05. It was proximal to genes *ERF113* and *HUB1*. Three SNPs (9A05_3320578, A05_3320584, A05_3320629) on chromosome A05 appeared associated with the gene *CYP76C3* encoding cytochrome p450 protein. SNP locus (A08_3147142) on chromosome A08, shared genome space with the gene *LECRK91* encoding Concanavalin A-like lectin protein kinase family protein. Chromosome A09 harboured three SNPs (A09_18223345, A09_18223348, A09_18223407) in a small genomic interval of 62 bp. These were present near to *RPS4*, *AT5G45060* and *AT4G19530* belonging to the disease resistance protein family (Fig. 5a). SNP locus (B05_13723719) on chromosome B05 was present close to the gene *ERF091* (Fig. 5b). Its protein product is known to be involved in positive regulation of JA responsive defence genes.

**Table 2:** The list of significant SNPs identified in consensus over seasons and different algorithms along with SNPs rich annotation information.

Chr	SNPs	SNP ID	Marker	PVE	−log10	SNPs consensus over		Annotation		Description
			Interval	(%)	(*p*)	Seasons	Algorithms	Gene Name	Gene Bank Identifier	
								(distance from SNP in kb)	(NCBI)	
A01	7	A01_11942966, A01_11943081, A01_11943121, A01_11944271, A01_11944321, A01_11944366, A01_11944403	11942966-4403	11.75	3.94	S1 + S2 + S3 + P	Farm CPU, GLM, MLM, GLM (T), MLM (T)	ACX1 (8.90)	332658399	Acyl-CoA oxidase 1
	1	A01_12606026	12606026	8.11	3.5	S1 + P	Farm CPU, GLM	LOX5 (4.50)	1032291524	Lipoxygenase-1
	1	A01_15152306	15152306	13.04	3.10	S2 + P	Farm CPU, GLM, GLM (T)	LOX1 (10.00)	1032297914	PLAT/LH2 domain-containing lipoxygenase family protein
	5	A01_23456622, A01_23456688, A01_23456778, A01_23456803, A01_23456875	23456622-875	8.25	3.1	S2 + S3 + P	Farm CPU, GLM, Adegnet	SOT 18 (15.00)	177666949	Desulfoglucosinolate sulfotransferase
	1	A01_23756215	23756215	8.18	3.35	S3 + P	Farm CPU, GLM	AT1G34047 (8.00)	240254195	Defensin-like (DEFL) family protein
A03	1	A03_7541673	7541673	11.52	3.21	S1 + P	Farm CPU, GLM, GLM (T)	phos32 (4.27)	75161512	Adenine nucleotide alpha hydrolases-like superfamily protein
								THE1 (0.80)	75335100	Malectin/receptor-like protein kinase family protein
A04	13	A04_19791849, A04_19791949, A04_19791988, A04_19791995, A04_19792000, A04_19792007, A04_19792025, A04_19792031, A04_19792043, A04_19792066, A04_19792069, A04_19792114, A04_19792116	19791849-2116	14.39	3.16	S3 + P	Farm CPU, GLM, GLM (T)	MPKKK17 (20.09)	15225692	Mitogen-activated protein kinase kinasekinase 15
A05	5	A05_2460823, A05_2460826, A05_2460835, A05_2460841, A05_2461061	2460823-1061	14.06	3.40	S3 + P	Farm CPU, GLM, GLM (T)			
								AT2G43620 (8.00)	15224321	Chitinase family protein
								CHI (5.84)	15224308	
	7	A05_3029963, A05_3030109, A05_3031956, A05_3049954, A05_3050015, A05_3050242, A05_3050244	3029963-50244	14.47	3.62	S2 + S3 + P	Farm CPU, GLM, MLM, GLM (T), MLM (T), Adegnet	ERF113 (18.00)	13272437	Ethylene responsive element binding factor 2 (ATERF2)
								HUB1 (3.77)	30689877	Histone mono-ubiquitination 2
	3	A05_3320578, A05_3320584, A05_3320629	3320578-629	16.04	3.69	S2 + P	Farm CPU, GLM, MLM, GLM (T), MLM (T), Adegnet	CYP76C3 (34.00)	330255479	Cytochrome P450, family 76, subfamily C, polypeptide 6
A08	1	A08_3147142	3147142	12.51	3.19	S3 + P	Farm CPU, GLM, GLM (T)	LECRK91 (13.79)	15238190	Concanavalin A-like lectin protein kinase family protein
A09	3	A09_18223345, A09_18223348, A09_18223407	18223345-407	15.53	3.75	S1 + S2	Farm CPU, GLM, GLM(T)	RPS4 (2.04)	15242354	Disease resistance protein (TIR-NBS-LRR class) family
								AT5G45060 (3.70)	15242300	
								AT4G19530 (4.10)	1063723668	
B05	1	B05_13723719	13723719	7.34	3.28	S2 + P	Farm CPU, GLM, MLM	ERF091 (4.94)	15233878	Ethylene responsive element binding factor 2 (ATERF2)

Figure in parenthesis indicate physical distance of the annotated gene from identified SNP.

### Linkage disequilibrium

The pairwise LD (*r*^2^) of significant and annotated SNP regions was analysed using software Haploview. LD blocks were identified on chromosomes A01, A04 and A05, which showed maximum MTA’s. Region in the LD blocks (black) indicated strong LD between pairs of SNPs (Supplementary Fig. S5). Pairwise LD estimation on chromosome A01 depicted the highest (1 kb) LD in block 1 region. Whereas, significant SNP region on chromosome A04 shows higher r^2^ value (black), followed by on chromosomes A01 and A05 with less than 1 kb of region.

### Validation of identified SNPs

We also compared a set each of susceptible and resistant lines to demonstrate polymorphism for associated SNPs. A majority of associated SNPs were also validated using whole genome sequences (21x) from five advanced generation lines (ADJR_8-Blue, ADJR_8-White, ADJR_8-Black, ADV_6-White and APH64), being bred for *Sclerotinia* resistance and three susceptible *B. juncea* germplasm lines (RLC1, NAJR-102B-R2 and DT1). Some of the identified SNPs were in heterozygous state. All this information is included as a supplementary data set (Supplementary Table S1).

## Discussion

Incorporation of resistance to *Sclerotinia* stem rot in a superior agronomic base is a major plant breeding goal in crop brassicas. Although the studies on *B. napus*^21,45^ and *B. oleracea*^17,46^ have previously helped to identify resistance related QTLs in A, B and C genomes of monogenomic and digenomic *Brassica* species, these could not be commercially exploited due to poor levels of resistance. While secondary and tertiary gene pools of *Brassicaceae* exhibit enormous genetic diversity against *S. sclerotiorum*^16^, the knowledge of putative gene candidates that contribute to basal/innate immunity remains scarce. *B. fruticulosa* is one of the wild relatives of *Brassica* that exhibits high resistance to this devastating pathogen. We successfully utilized next generation strategies of allele mining and candidate gene identification through SNP based approach to characterize genomic introgressions of *B. fruticulosa* in order to identify potential candidates for stem rot resistance. This study is significant as it mapped 13 significant loci on seven chromosomes (A01, A03, A04, A05, A08, A09 and B05). In addition, annotation of the genomic region around identified SNPs allowed prediction of 20 candidate genes belonging to major disease resistance protein families, including TIR-NBS-LRR class, Chitinase, Malectin/receptor-like protein kinase, defensin-like (DEFL), desulfoglucosinolate sulfotransferase protein and lipoxygenase. Importantly, our findings not only provide critical new understanding of the defensive pathway of *B. fruticulosa* resistance, but will also enable development of marker candidates for assisted transfer of introgressed resistant loci into agronomically superior cultivars of crop *Brassica*.

**Figure 6:**
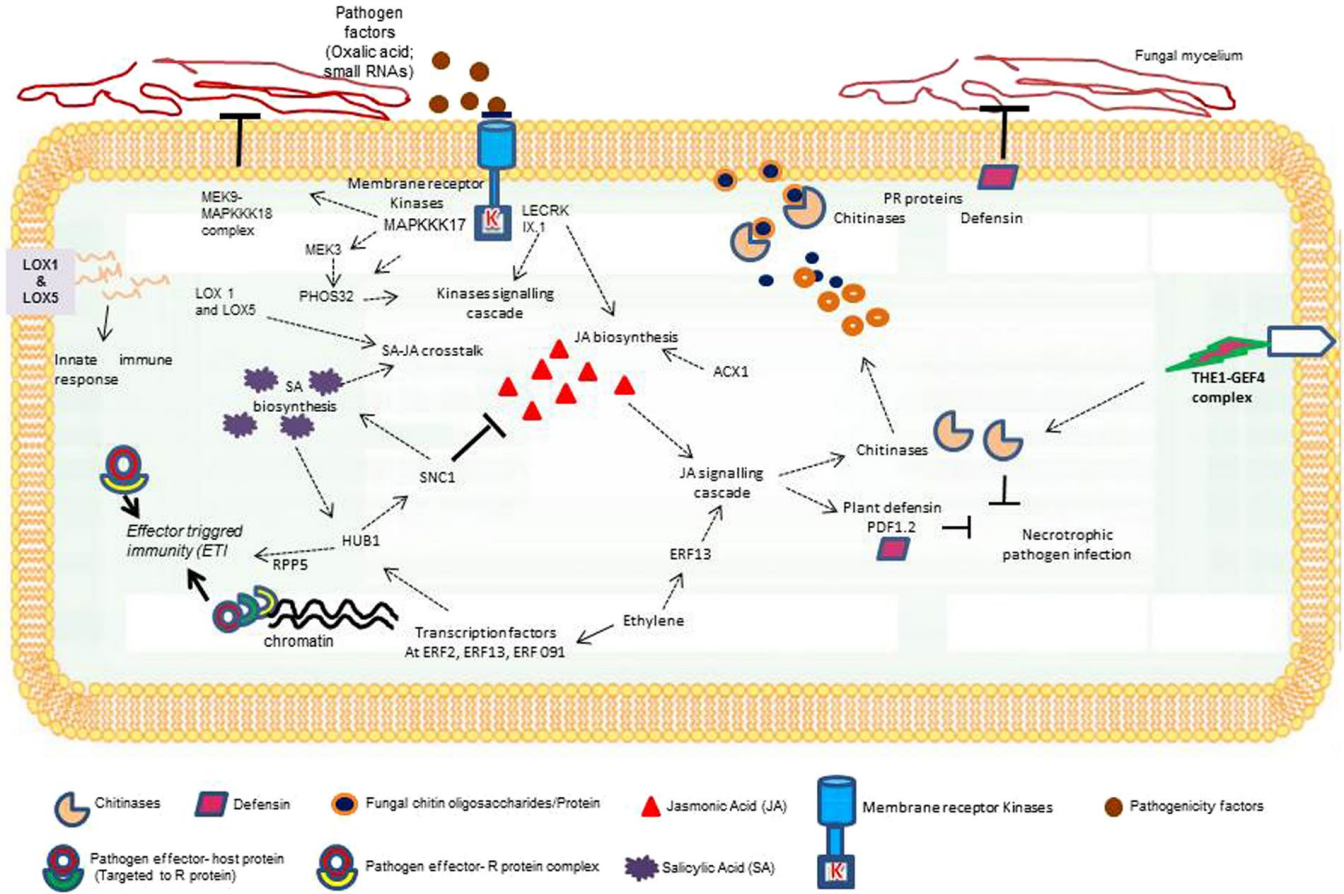
Proposed model to describe transcription factors association and phytohormones crosstalk in resistance responses against *Sclerotinia sclerotiorum* in *Brassica juncea* – *B. fruticulosa* introgression lines. *ACX1*: Acyl-CoA oxidase 1, *ERF*: Ethylene response factor, *GEF4*: Guanine exchange factor4, *HUB1*: Histone mono-ubiquitination 1, JA: Jasmonic acid, *LOX*: Lipoxygenase, *LECRK91*: L-type lectin receptor-like kinase IX.1, *MAPKKK*: Mitogen-activated protein kinase 17, *MEK*: *MAPK* kinases, *PDF1.2*: Plant Defensin 1.2, PR: Pathogenesis related protein, *RPP5*: Resistance to *Peronospora parasitica5*, SA: Salicylic acid, *SNC1*: Suppressor of npr1-1, constitutive1, *THE1*: THESEUS1.

A well-orchestrated network involving diverse defence signalling cascades may arise during plant-pathogen interaction in stem rot (Fig. 6). It initiates with pathogen associated molecular patterns (PAMPs) perceived at host surface; through switching on of PAMP triggered immunity (PTI) via receptor like kinases (RLKs). But if this basal non-race-specific immune response is surpassed by host adapted pathogens, effector triggered immunity (ETI) sets in via release of R proteins (such as *SNC1*, *RPP4*). Tight regulation of gene expression for fine tuning defensive responses is necessary, otherwise it may impair the fitness of the host plant^47,48^. Such reprogramming of immune response is either mediated by pathogen infection or through intracellular signals whose downstream players alter gene expression through chromatin remodelling^19^, or transcriptional or post translational modification. *HUB1*, located close to 3 SNPs, identified on chromosome A05, is one such novel gene that is responsible for histone modifications (*i.e*., methylation and deacetylation). This is a large effect QTL, explaining 14.5% of the phenotypic variation. Mutant studies have revealed that histone 2B monoubiquitinylation through *HUB1* activity induces expression of various R genes/R gene complex including *SNC1*, *RPP5* in *Arabidopsis* against necrotrophic fungi like *Botrytiscineria* and *Alternaria brassicicola*^49,50^. Upstream regulation of *HUB1* by hormonal signals (ethylene and salicylic acid) controls chromatin architecture of *SUPPRESSOR OF NPR1-1* and *CONSTITUTIVE1* (*SNC1*). These are key regulators of jasmonate-salicylate (JA-SA) crosstalk during systemic and local defences. We found association of 3SNPs on A05 (*ERF13*) and 1SNP on B05 (*ERF 91*) with *ATERF2* family. *ATERF2* are plant specific transcription factors (TFs) that belong to B3 sub-cluster of APETALA2/ethylene response factor (*AP2*/*ERF*) family. They play a crucial role in transcriptional regulation of JA-ethylene (ET) responsive defence genes (such as *PLANT DEFENSIN1.2*/*PDF1.2; BASIC CHITINASES/CHI B/PR3*) and thus integrate the hormonal crosstalk against *necrotrophic* pathogens^51–53^. Association of *ERF13* with *Sclerotinia* stem rot resistance in the present study is supported by previous reports on its overexpression with MeJA^51^ and *A.brassicicola*^51^ and chitin^54^ in *Arabidopsis* for transcriptional activation of JA-induced disease resistance.

Aside, TFs an arsenal of cell surface-localized pattern recognition receptors (*PRR*) with intracellular kinase domain also fine tune the temporal responses through expression of defensive genes in pathogen-challenged plants. We identified 15SNPs, located to chromosomes A03, A04 and A08 that were strongly associated with *Sclerotinia* resistance in ILs through kinase signalling cascade. Out of these, 13SNPs formed an LD block on A04 close to the gene encoding mitogen-activated protein kinase, *MAPKKK17*. However, SNPs corresponding to other receptor kinases *LECRK IX.1*/*LECRK91* and *THESEUS1* (*THE1*) on chromosomes A08 and A03, were also observed. These contributed up to 24.03% of phenotypic variation for disease resistance. Transcriptome studies in *B. napus*^55,56^ have emphasized the role of *BnaMAPKKK17* (an orthologue of *AtMAPKKK17*), *Bna MAPKKK18* and its downstream associated kinases (*MKK3*, *MKK8* and *MKK9*) in abiotic and biotic stress tolerance. *THE1*, a CrRLK1 like kinase may sense cell wall integrity and mediate defence against necrotrophic fungi like *B. cinerea* in *Arabidopsis* through elicitation of *GEF4* signalling network^57^. Further, the overexpression of *THE1* caused increased deposition of callose, H_2_O_2_ accumulation and also the upregulation of *PR2* and *PDF1.2* transcript levels in *B. cinerea* treated plants as compared with the wild type^57^. Studies on *LECRK91*^58,59^ have addressed their role in plant innate immunity through cell wall associated defences and jasmonate signalling. Mutation in *LECRK91* caused susceptibility to fungal and bacterial pathogens in *Arabidopsis*, while its overexpression reversed the effect, thereby enhancing immune response by cell death^59,60^.

In our ILs, 7SNPs in a genomic interval of 1.4Kb on A01 appeared very close to the gene encoding Acyl–CoA oxidase (*ACX1*) that has a role in JA biosynthesis. These explained 11.75% of phenotypic variation. Three additional SNP markers on chromosome A01 and A03 appeared associated with lipoxygenase *LOX1*, *LOX5* and hydrolases *PHOS32*. *In vitro* assays have confirmed phosphorylation of *PHOS32* by *AtMPK3* and *AtMPK3* upon elicitation; and suggested its role in mediating downstream kinase signalling events^61^. Both *LOX1* and *LOX5* encoding 9-lipoxygenases, were responsible for hydro-peroxidation of fatty acid component of plant membranes. Increased activity of lipoxygenase has already been reported in a number of plant pathogen systems^62,63^, including *Arabidopsis*^64^, where loss of function of *LOX1* and *LOX5* mediated resistance occurs against *Fusarium* head blight. Their hormonal regulation with JA, salicylate (SA) in addition to other stress like wounding suggests that LOX control balance of JA-SA signalling. We observed a major locus with large effect (14.06% phenotypic variation) on chromosome A05, with significant SNPs near to candidate gene of the Chitinase family. Chitinases, also known as pathogenesis related (PR) proteins, are generally induced in the plant system in response to fungal, bacterial or viral infections. The chitinase genes *At2g43620* and *CHI* belong to class IV chitinases and are involved in cell wall biogenesis. These are induced upon pathogen infection^65,66^ and hormones like ethylene^67^. We also identified a MTA (A01_23756215) close to another PR protein, defensin. Inductions of these defensins or defensin-like proteins represent a host innate immune response which is triggered upon pathogen infection. These cysteine rich cationic peptides modify fungal membrane permeability and inhibit growth of a broad range of fungal pathogens^68^.

Overexpression of defensin genes in transgenic *B. napus* resulted in enhanced resistance to *S. sclerotiorum*^69^. Me-JA and SA application reveals complex networking between *PR* genes and hormonal signals as indicated by their induction in response to biotic stimuli. Identification of genomic region, harbouring 3SNP markers on A09, in proximity of TIR-NBS-LRR class of resistance genes (*RPS4*; *AT5G45250*) within a 62Kb region, seemed important. R genes like *RPS4* recognise fungal effector-host protein complex and activate ETI against both fungal and bacterial pathogens. *RPS4*, alone or in pair with *RRS1* (R protein complex), can act as a dual resistance system against the hemibiotrophic fungus *Colletotrichum higginsianum* in *Arabidopsis*^70^. Downstream signalling after activation of TIR-NBS-LRR involves genes *EDS1* and *EDS1* related proteins (PAD4 and SAG 101) with JA-SA regulation of immune responses. Present study also confirmed the role of *NIP3-1* as reported previously^18^. This locus was in strong LD with the SNP A01_ 12606026. Thus search of putative candidates through systematic annotation in identified genomic region following GWAS helped us to identify genomic regions that likely contributed to *Sclerotinia* resistance in *B. juncea*-*B. fruticulosa* ILs. A majority of the significant SNPs could be validated using whole genome sequences (21x) from five advanced generation lines being bred for *Sclerotinia* resistance as compared to three susceptible *B. juncea* germplasm lines. We now hope to develop breeder friendly KASPar markers for effective integration of these defensive components into a superior agronomic base, initially in *B. juncea* and subsequently in *B. napus* and other vegetable *Brassica* species.

## Methods

### Plant materials and resistance screening

Genetic materials and stem inoculation procedures were same as described earlier^18^. Eighty eight ILs were raised in a randomized block design with two replications over three crop seasons against an aggressive and virulent strain (PAU-4) of *S. sclerotiorum* at Punjab Agricultural University, Ludhiana, India. Analysis of variance (ANOVA) was undertaken and standard deviation and standard errors were estimated through Multivariate General Linear Model (M-GLM) using software, SPSS17.0.

### DNA extraction for library preparation

Genomic DNA of ILs and their parents were isolated from young leaf tissue using a CTAB (cetyltrimethyl ammonium bromide) extraction method^71^, with the chloroform-isoamyl alcohol purification step repeated twice to assure good quality DNA. RNase was added between the two chloroform: isoamyl alcohol solvent extractions by incubating the samples at 37° Celsius for 15 minutes to allow for a single DNA precipitation step at the end of the protocol. Quality of the extracted DNA was assessed using a NanoDrop® 2000 spectrophotometer and 1% (w/v) agarose gel. Sample DNA having 260/280 absorbance ratio of 1.8-2.0, and with no evidence of substantial band shearing or contamination (either RNA or polysaccharide), was used for library construction.

### Resequencing and genotyping by sequencing

Direct resequencing (21x) of four ILs and genotyping by sequencing of 88 ILs were outsourced. For this, high quality DNA of each sample was digested with appropriate combination of restriction enzymes based on in-silico evaluation. This was followed by several rounds of PCR amplification. All samples were then individually pooled and then size-selected for the required fragments to complete the library construction. High quality libraries with appropriate insert sizes were subsequently used for pair-end sequencing on Illumina® HiSeq platform, with the read length of 150 bp at each end. The sequences and corresponding sequencing quality information were stored in a FASTQ file format. The adapter sequences were removed from raw reads using the software Cutadapt. The available reference genome of *B. juncea* v1.5 was used for reference-based alignments of whole genome sequences (21x) from the four most prominent ILs, using software bowtie2. Initially, one introgression line was aligned into reference genome and SNP called by NGSEP-GBS pipeline. Total SNPs with base quality more 30 were replaced in background genome reference (*B. juncea* v1.5) using a perl script, pseudomaker.pl implemented in SEG-Map^72^ to construct the first step of mock-up pseudomolecule(s), which were then used as a reference for subsequent construction of pseudomolecules of sequential replacement of SNPs for remaining three ILs in the same way as described above.

Resultant mock-up reference was then used for aligning sequence tags from 88 ILs. SNPs identification was then carried out by using NGSEP-GBS pipeline^73^. Resultant marker dataset comprised 31,896,811 SNPs. These were then filtered to include only quality SNPs for further analysis. Filtering parameters were: minimum mapping quality (30), minor allele frequency (0.08), bi-allelic SNVs, minimum number of sample genotyped (70), maximum observed heterozygosity (30%) and maximum missing calls (30%). After filtering, 88,624 SNPs remained. These were imputed using fcGENE and Beagle softwares^74^.

### Genome-wide association analysis (GWAS) based on SNP genotyping

*Sclerotinia* resistance lesion length (cm) data were first normalized through rank and log transformation. GWAS was carried out using transformed values of lesion lengths and 88,624 SNPs. Kinship matrix and Covariates data was generated through ‘MVP.Data’ function of the software R (MVP-package). Discriminant Analysis of Principal Components (DAPC) was implemented in R software package “adegenet”. PC and DAPC values were then used as covariates in different GWAS analysis algorithms to reduce false positives by accounting for the effects of population stratification. Manhattan plots were generated with multi-model plotting using MVP tools. GLM, MLM and FarmCPU methods were implemented in the software R (MVPpackage) (https://github.com/XiaoleiLiuBio/MVP), using default settings to identify marker trait associations. Identified markers were subsequently confirmed using alternate algorithms as implemented in TASSEL5.0 with DAPC (in adegenet) as covariate for GWAS with model; GLM + DAPC [GLM(T)] and MLM + kinship [MLM(T)]. Visualization of the significant QTLs and SNPs was done using Manhattan plots, generated using the R package qqman^75^. We used unadjusted p-values < 0.001, to declare significance as the Bonferroni and FDR tests are considered far too conservative for association studies involving thousands of candidate variants at very low minor allele frequencies^76^. Bonferroni correction also assumes independence of every association test, an assumption which is rarely met due to linkage disequilibrium between SNP markers. We screened 50 kb flanking regions on each side of resistance associated peak SNPs to predict candidate genes using Blast2GO v5.2.5 tool (Gotz^77^ *et al*. 2008). *Arabidopsis* protein database was probed for gene finding as well as blast search. The gene ontologies of *Arabidopsis* orthologues were used for this analysis as these are better curated than those of *B. juncea*. Positions of the predicted candidate genes w.r.t. the SNPs, were projected through blast search of their sequences against *B. juncea* mock-up pseudomolecules. Predicted candidate genes were then verified for their function from literature for their relevance to the target trait.

## Supplementary information

Supplementary Fig. S1-5

Supplementary Table S1.
