# Supplementary material for: Molecular and genetic analysis of defensive responses of Brassica juncea – B. fruticulosa introgression lines to Sclerotinia infection
Source: Sci Rep. 2019 Nov 19;9:17089. doi: 10.1038/s41598-019-53444-3 (PMC6864084; doi:10.1038/s41598-019-53444-3)
Supplement: Supplementary file 1 — Supplementary Fig. S1-5 [file 41598_2019_53444_MOESM1_ESM.pdf]

Molecular and genetic analysis of defensive responses of *Brassica juncea – B. fruticulosa* introgression lines to Sclerotinia infection

Chhaya Atri[1], Javed Akhatar[1], Mehak Gupta[1], Neha Gupta[1], Anna Goyal[1], Kusum Rana[1], Rimaljeet Kaur[1], Meenakshi Mittal[1], Anju Sharma[1], Mohini Prabha Singh[1], Prabhjodh S. Sandhu[1], DBT Centre of Excellence on Brassicas, Department of Plant Breeding and Genetics, Punjab Agricultural University, Ludhiana -141004, Punjab, India; **Martin J. Barbetti[2]** School of Agriculture and Environment and the UWA Institute of Agriculture, Faculty of Science, The University of Western Australia, 35 Stirling Highway, Crawley, WA, 6009, Australia; and **Surinder S. Banga[1]**, DBT Centre of Excellence on Brassicas, Department of Plant Breeding and Genetics, Punjab Agricultural University, Ludhiana-141004, Punjab, India.

*Corresponding author: S. S. Banga; E-mail address: nppbg@pau.edu

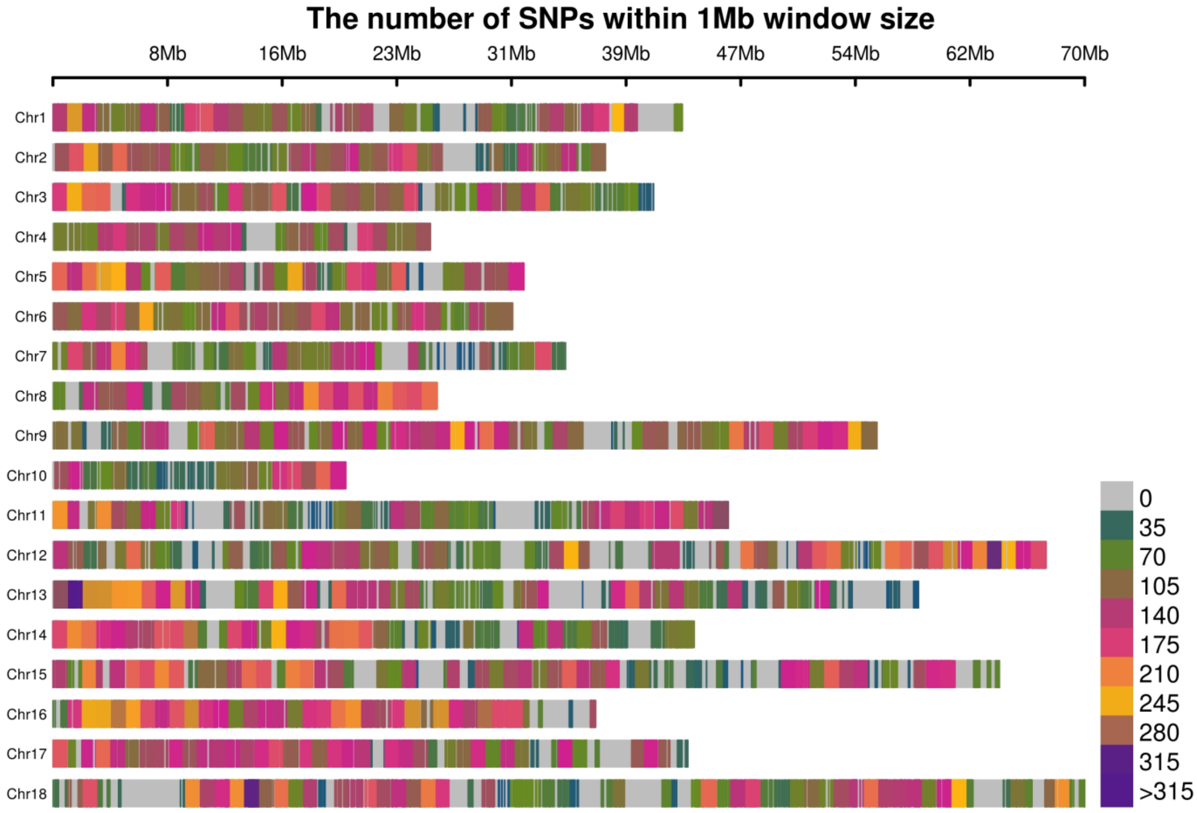

Supplementary Fig. S1: Graphical representation of SNP density on 18 chromosomes of *Brassica juncea*.

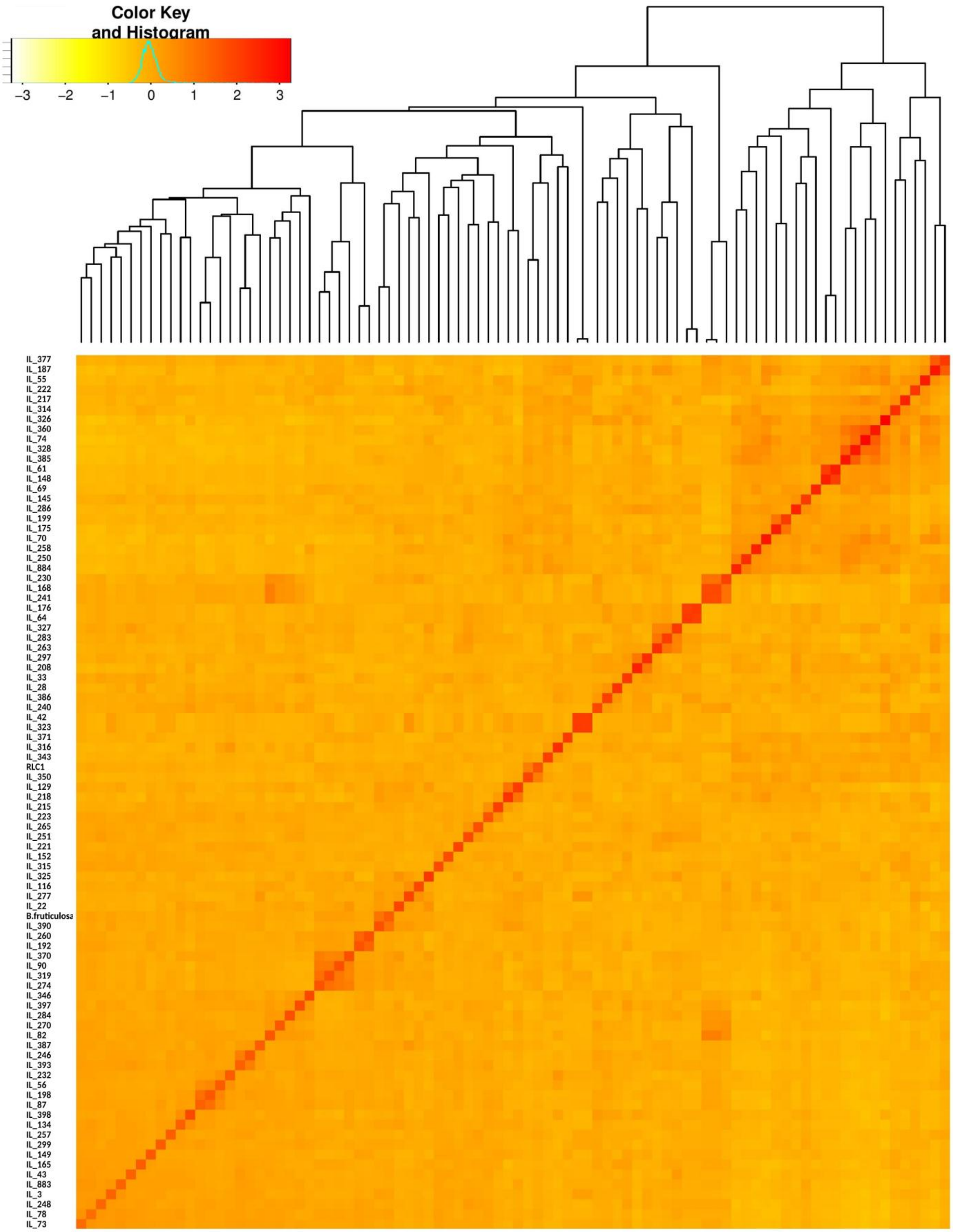

Supplementary Fig. S2: Heatmap and clustering of kinship matrix generated by 88,624 high quality SNPs.

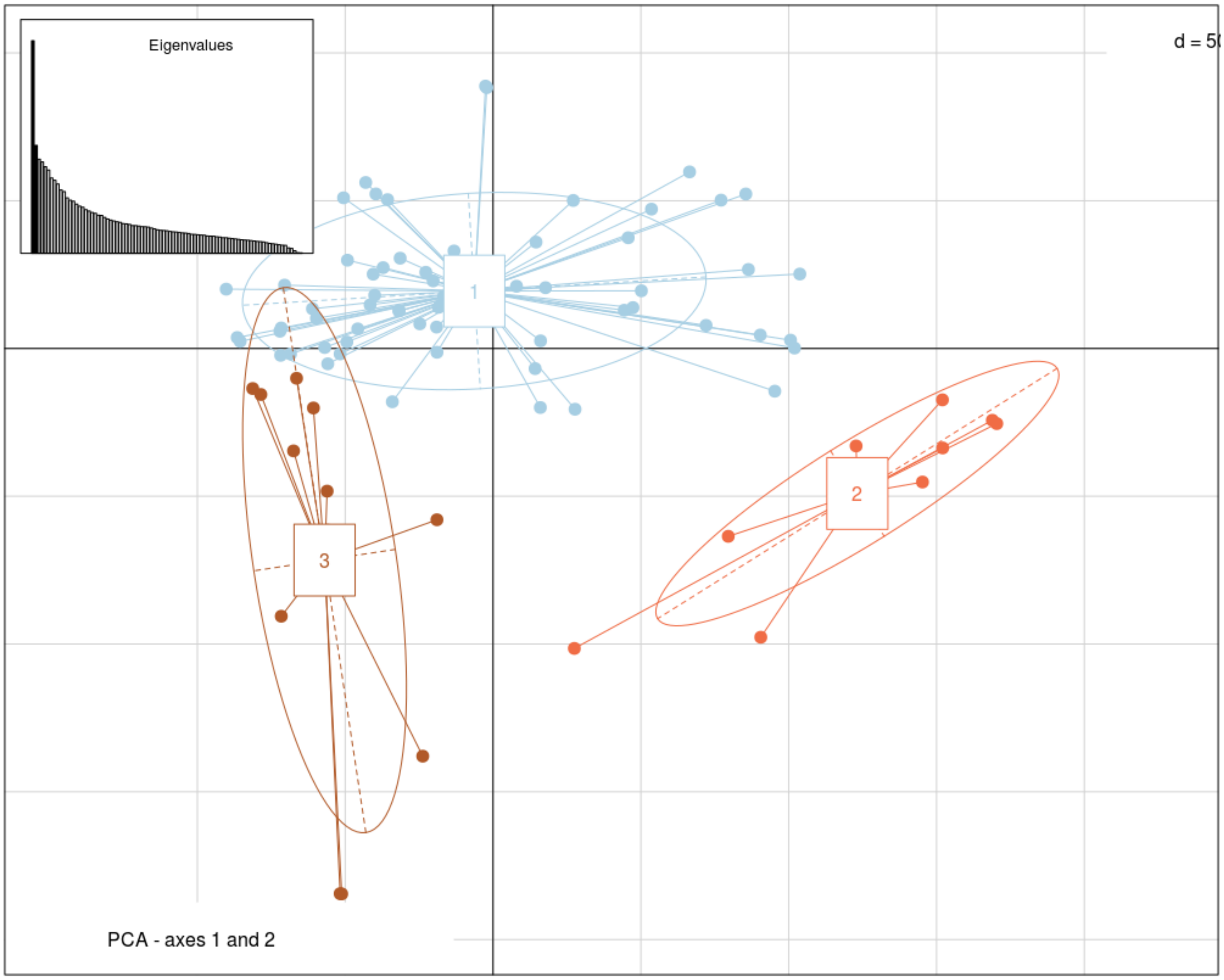

Supplementary Fig. S3: Genetic Discriminant Analysis of Principal Components (DAPC) analysis of 88 *Brassica juncea* – *B. fruticulosa* introgression lines.

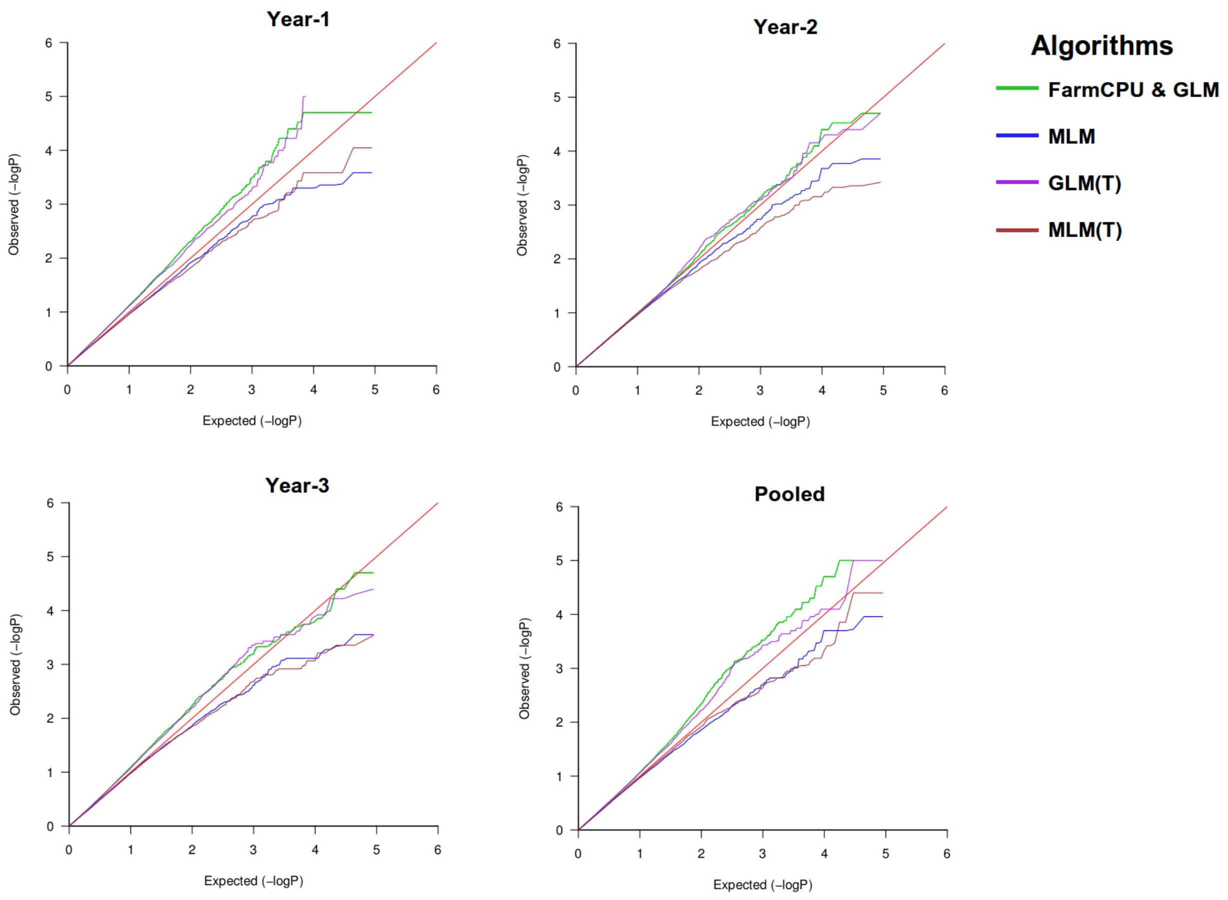

Supplementary Fig. S4: QQ plots for final model fitting.

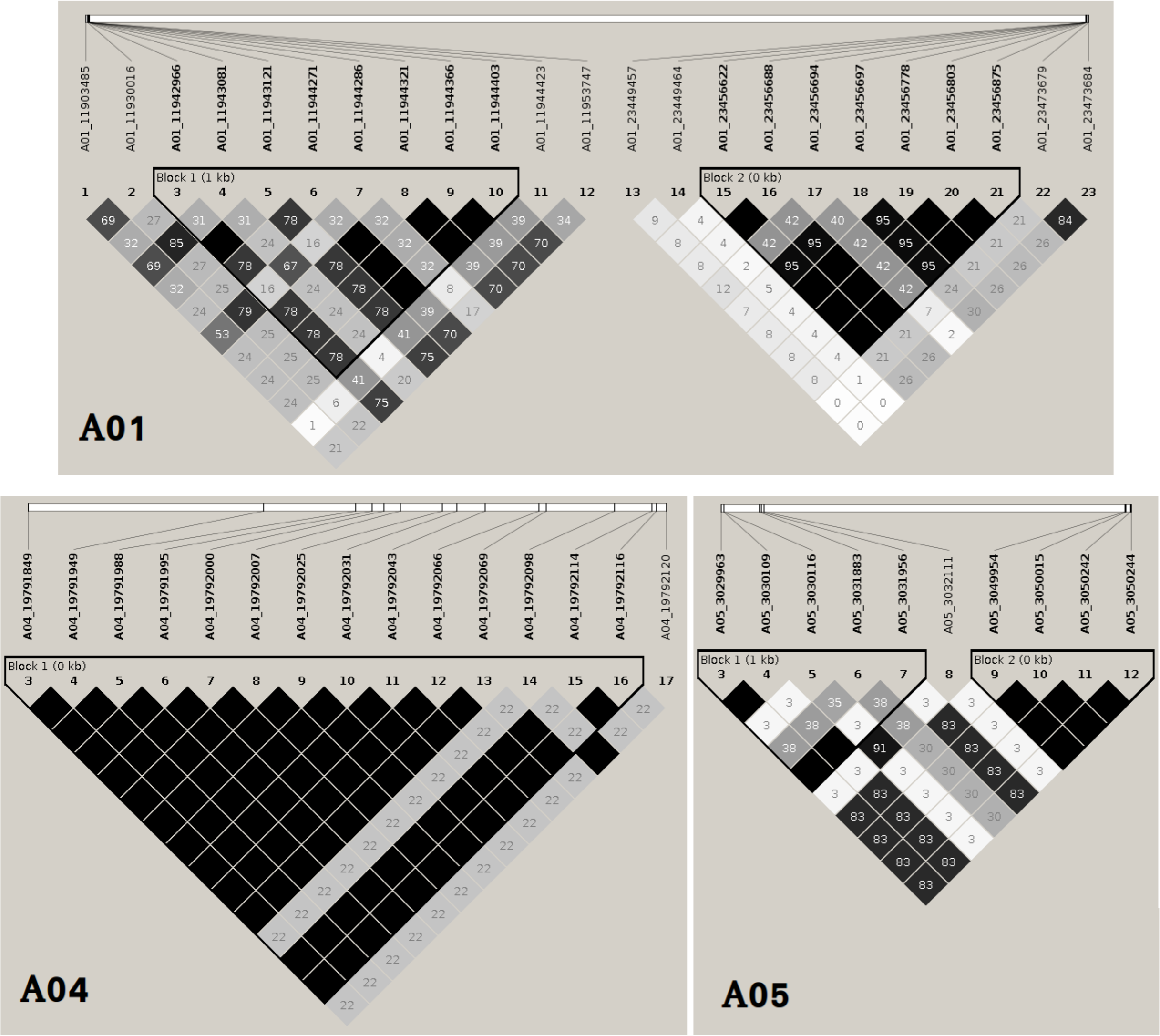

Supplementary Fig. S5: Haploview LD plot of significant SNPS on chromosomes A01, A04 and A05.
